# Review of fruit cork spot disorder of Asian pear (*Pyrus* spp.)

**DOI:** 10.3389/fpls.2023.1211451

**Published:** 2023-06-29

**Authors:** Xiaozhen Zhang, Zhenhua Cui

**Affiliations:** ^1^Department of Agriculture and Forestry Science and Technology, Lanzhou Modern Vocational College, Lanzhou, Gansu, China; ^2^Department of Horticulture, Qingdao Agricultural University, Qingdao, Shandong, China

**Keywords:** Asian pear, cork spot disorder, symptoms, causal factors, preventive measures

## Abstract

Cork spot disorder has affected the fruit of Asian pear since the 1990s and has become serious in recent years with increasingly affected cultivars and areas. The commodity value of affected fruit is greatly decreased, resulting in severe economic losses. Cork spot disorder of pear fruit is a physiological disorder, and the factors responsible are relatively complex. Research on the cause of cork spot disorder is still at an early stage and, thus, further investigations are needed to elucidate the underlying mechanism of the disorder. In this review, current knowledge of the factors associated with the incidence of cork spot disorder in Asian pear fruit is summarized, including fruit growth and development, fruit nutrient status, and environmental factors. Potential preventive measures and priorities for future research are outlined.

## 1 Introduction

Pears (*Pyrus* spp.) are widely cultivated in Asia, among which the Asian pear is especially favored by consumers. In recent years, new pear cultivars and novel cultivation techniques for pear trees have been promoted and rapidly popularized. However, many new cultivars lack adaptability to climatic stresses, which has resulted in an upsurge in the incidence of physiological disorders of the fruit, such as watercore, hard end, and cork spot disorder (Kajiura et al., 1976; Tanabe et al., 1982; Yamamoto and Watanabe, 1982; Tamura et al., 2003; Nakamura, 2011; Ma et al., 2014; Duan et al., 2019; Cui et al., 2020). These fruit physiological disorders pose novel challenges for Asian pear production, especially cork spot disorder, which is prevalent in certain Asian pear cultivars, decreases the fruit commodity value, counterbalances the favorable traits of some new cultivars, limits the utilization of some new cultivars, and causes major losses to pear production (Nakamura, 2011; Hayama et al., 2017; Tamura, 2017; Duan et al., 2019; Cui et al., 2020). Preliminary investigations of the characteristics of cork-spotted fruit have been reported, including the development period of cork spot disorder, the flesh tissue structure and nutrient metabolism of affected fruit, and hormonal regulation (Nakamura, 2011; Mitani et al., 2017). Experiments have been conducted, involving soil improvement, regulation of tree growth, and chemical treatment, to explore effective preventive measures, which have achieved some positive outcomes (Hayama et al., 2017; Duan et al., 2019). Despite considerable investment in studying the prevention and control of cork spot disorder, the mechanism of initiation of cork spot disorder remains uncertain, which limits the application of effective preventive countermeasures. Therefore, there is an urgent need for research into the mechanism of initiation of cork spot disorder and the development of preventive measures. In this review, with consideration of the results of field-based investigations and in the laboratory on cork spot disorder in recent years, we summarize progress in understanding the characteristics, development, and prevention of cork spot disorder and suggest ideas for future research on cork spot disorder of pear fruit.

## 2 Symptoms of cork spot disorder in pear fruit

**Figure 1:**
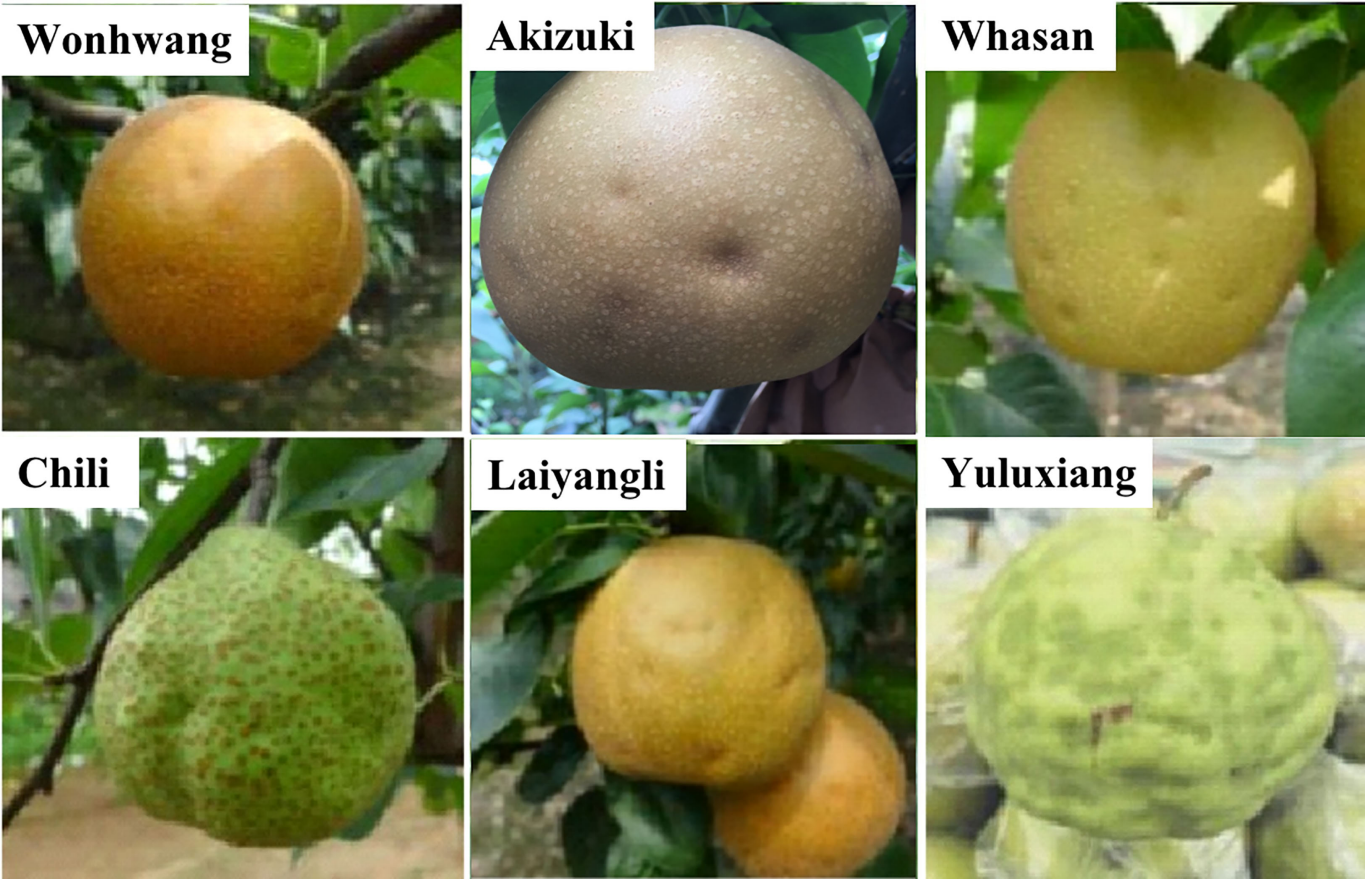
Symptoms of cork spot disorder in Asian pear fruit in the field. The upper row are cultivars of sand pear (*Pyrus pyrifolia*), and the lower row are cultivars of white pear (*Pyrus bretschneideri*).

The main symptoms of cork spot disorder in pear fruit are the appearance of round, sunken spots in the fruit skin, browning and lignification of the flesh under the sunken area, desiccated tissue in the vicinity of vascular bundles, and production of bitter substances in the flesh in severe cases (McAlpine, 1921; Nakamura, 2011; Tamura, 2017). The symptoms are prone to develop in the outer flesh and in proximity to the equator of the fruit (Duan et al., 2019; Cui et al., 2020). In addition, the symptoms differ among pear cultivars. Among cultivars of Chinese white pear (*Pyrus bretschneideri*), in ‘Chili’ cork spot, symptoms appear at ~80 days after full bloom (DAFB) with the development of bumpy, irregular protrusions in the fruit skin (**Figure 1**). In contrast to ‘Chili’, ‘Yuluxiang’ fruit develops more severe cork spot symptoms with a high density of sunken areas in the skin (**Figure 1**). Among sand pear (*Pyrus pyrifolia*) cultivars, the cork spot symptoms of ‘Akizuki’, ‘Whasan’, and ‘Wonhuwang’ mainly comprise circular sunken spots on the fruit surface without other visible symptoms (**Figure 1**). The symptoms in sand pear cultivars appear relatively later during fruit development than in white pear cultivars. Differences in fruit developmental phases and flesh tissue structure may contribute to the variation in symptoms of cork spot disorder among pear cultivars.

**Figure 2:**
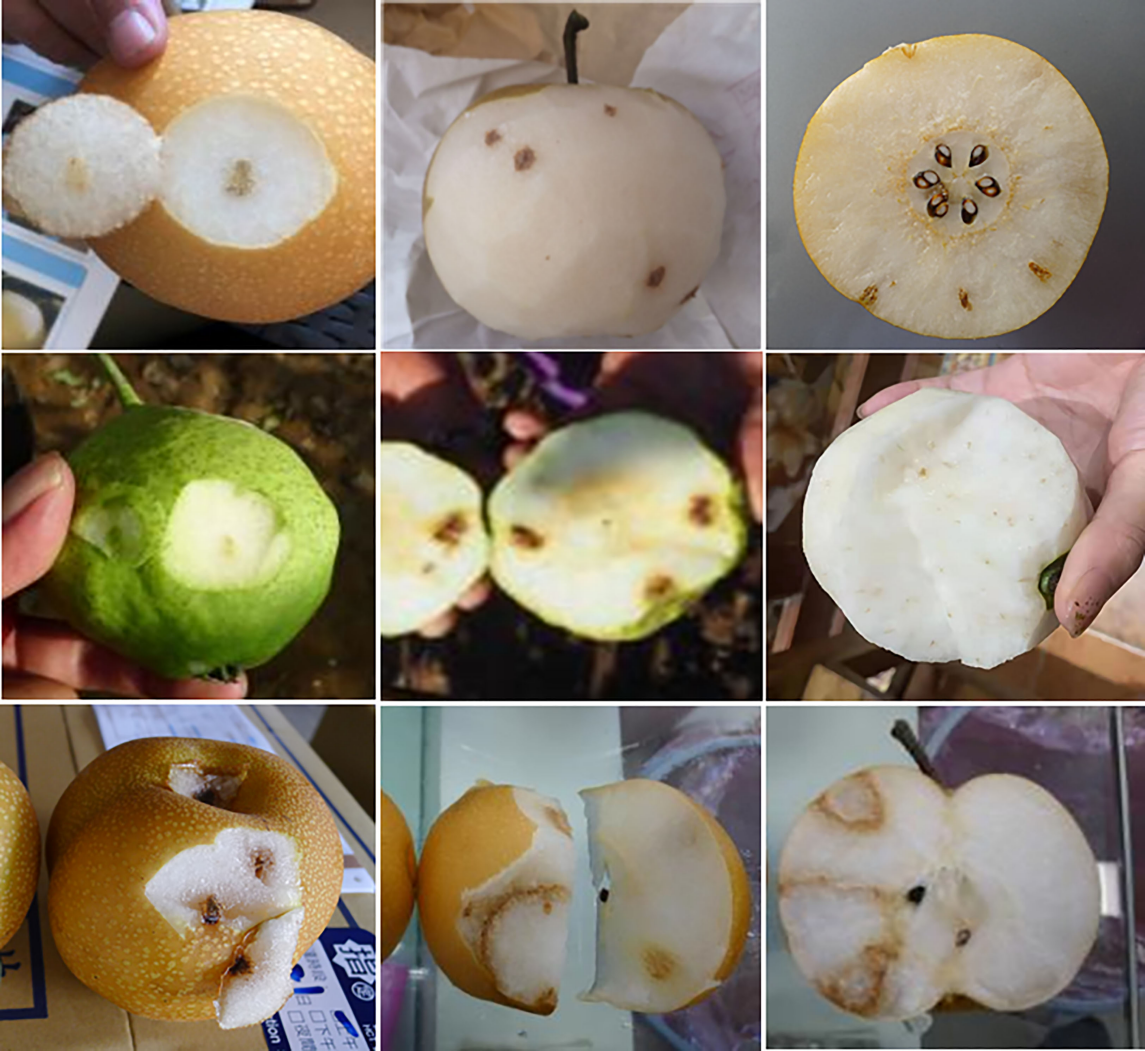
Symptoms of cork spot disorder in the fruit flesh of different cultivars of Asian pear.

With regard to internal symptoms of cork spot disorder in the fruit flesh, three types of symptoms are observed (**Figure 2**). First, circular sunken areas develop in the fruit skin, gradually extending into the underlying flesh, and form cylindrical spots in the flesh, which is similar to the symptoms of bitter pit in apple (*Malus* × *domestica*). Rarely, these symptoms are observed in the flesh in the vicinity of the fruit core. Second, browning and lignified circular spots develop in the outer flesh. The third type of symptom develops in the fruit pericarp with a radiate, filamentous distribution. Browning and necrotic spots are scattered in the flesh, but no symptoms are visible on the fruit surface. The first symptom type is the most commonly observed, whereas the other two types are observed less frequently in comparison.

## 3 Factors affecting the incidence of pear cork spot disorder

### 3.1 Cultivar susceptibility

Based on the results of previous investigations, cork spot disorder in Asian pears is mainly reported in recently bred cultivars, such as ‘Akizuki’, ‘Oushuu’, ‘Xueqing’, ‘Yuluxiang’, ‘Xinli7’, and ‘Zaosu’ (Nakamura, 2011; Duan et al., 2019; Cui et al., 2020). The incidence of cork spot disorder is much lower in ‘traditional’ cultivars, such as the Chinese pear cultivars ‘Yali’, ‘Xuehuali’, and ‘Korla Pear’ (Wang et al., 1990; Dong et al., 2018). The long-term adaptability of traditional cultivars to diverse environmental stresses may contribute to the lower incidence of cork spot disorder. By contrast, a high fruit sugar content was a focus in the development of recently bred cultivars, but evaluation of their adaptability to environmental stresses was deficient, resulting in the increased susceptibility to cork spot disorder. Therefore, cork spot disorder tends to be most frequent in cultivars with fruit of otherwise excellent quality, especially a high sugar content. In future, tolerance of environmental stresses should be evaluated carefully during the breeding of new cultivars, which may avoid the economic losses resulting from inadequate assessment of the adaptability of new cultivars before their release.

### 3.2 Fruit growth and development

Some previous studies have reported that cork spot disorder is significantly more frequent in large fruit than in small fruit in ‘Akizuki’. Thus, the occurrence of cork spot disorder is strongly associated with fruit growth and development, especially fruit enlargement. Cell division in the early stages of fruit development determines the total cell number in the fruit and the fruit growth rate in the enlargement phase. To improve cell division in immature fruit, the balance between shoot growth and fruit growth should be manipulated to minimize the influence on fruit growth of competition for nutrients from the branches and leaves (Tamura, 2017). Cell division in the fruit can be promoted by fruit thinning as early as possible to increase the number of cells in the remaining fruit, which effectively prevents the development of excessively large cells during fruit enlargement and reduces the incidence of cork spot disorder. The growth vigor of the tree can be controlled by adjusting the angle of the branches and by fertilizer application, which effectively reduce competition for resources between shoot growth and fruit growth. In addition, large fruit generally develop in an advantageous position on the tree body and accordingly will have a strong demand for nutrients and be sensitive to nutrient supply. Therefore, a stable nutrient supply is important to maintain stable growth of large fruit and to reduce the incidence of cork spot disorder in large fruit.

### 3.3 Flesh tissue structure

**Figure 3:**
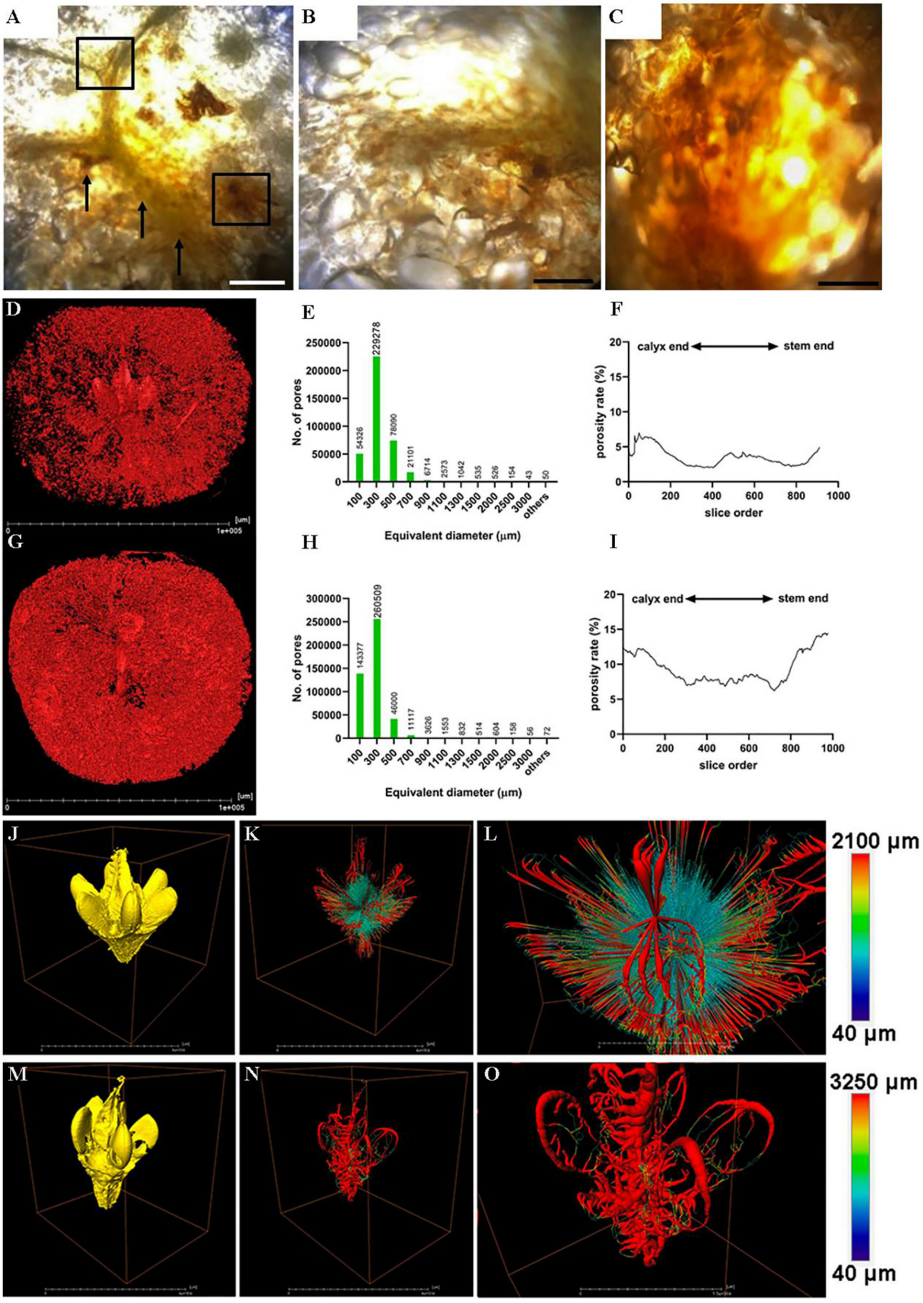
Different ways of observation of the flesh structure of cork spotted pear fruit summarized from Cui et al. (2020) and Cui et al. (2021a). **(A–C)** are the anatomic observation of the cork spotted flesh tissues. Brown necrotic tissues on the vascular bundles were marked by black arrows in **(A)**; **(B, C)** are the closer views of the squared area of A (upper left and lower right, respectively). **(D–I)** are the microstructural analysis of the whole fruit with or without cork spot disorder by X-ray CT scanning. **(D, G)** are the 3D renderings of X-ray scanning of healthy and cork spotted fruit, respectively. Red dots represent pores based on the grayscale value threshold; **(E, H)** are histograms of the pore number and size in healthy and cork spotted fruit, respectively; **(F, I)** are porosity analysis of healthy and cork spotted fruit, respectively. The segmentation of the X-ray scanning data were conducted from the calyx end to the stem end of the fruit. **(J–O)** are the structure and network analysis of the fruit core with or without cork spot disorder by X-ray scanning and 3D-rendered techniques. **(J, M)** are reconstructed 3D models of the healthy fruit core and cork spotted fruit core, respectively; **(K, N)** are reconstructed 3D network models of **(J, M)**, respectively; **(L, O)** are closer views of K and N, respectively. Color bars on the right indicate pore thickness from narrow (blue) to wide (red).

Even though cork spot caused visible sunken spots on the fruit surface, no association between the characteristic alteration of the peel and the occurrence of cork spot disorder has been found. Histological observations of cork-spotted fruit indicate that cork spots mainly appear in the pericarp and rarely develop in tissues in proximity to the fruit core. Cork spots initially appear near vascular bundles in the fruit and gradually expand in extent (Nakamura, 2011; Duan et al., 2019). These results indicate that cork spot disorder is closely associated with the functions of vascular bundles in the flesh, which are mainly responsible for the transport of water and nutrients in the fruit. Application of X-ray computed tomography (X-ray CT) scanning to analyze the three-dimensional (3D) pore structure of ‘Akizuki’ fruit revealed that the porosity of cork-spotted fruit (9.37%) was significantly higher than that of non-affected fruit (3.52%). In addition, the pore channels of cork-spotted fruit were highly branched and the degree of pore connectivity was much higher than that of non-affected fruit (Duan et al., 2020; Cui et al., 2021a). These findings indicate that the flesh tissue structure is changed significantly, the cell space and porosity of the flesh tissue are increased, and the overall flesh exhibits a loose texture and ultimately assumes a more highly porous microstructure in cork-spotted fruit, as summarized in **Figure 3**. Dysfunction of the vascular bundles disrupts the balance of water and nutrient transport within the fruit, which may account for alteration of the pore structure in the flesh of cork-spotted fruit.

### 3.4 Mineral nutrients

#### 3.4.1 Calcium

Calcium (Ca) is an essential nutrient for fruit growth and development and plays important structural roles in flesh cells. Calcium deficiency is considered to be associated with physiological disorders of the fruit in many plant species (Raese, 1988; Raese and Drake, 1995; Lee et al., 2007; Miqueloto et al., 2014; Dong et al., 2015). Symptoms of cork spot disorder of pear fruit are similar to those of apple bitter pit, which has been proven to be associated with Ca deficiency. Therefore, it is considered that incidence of cork spot disorder is mainly caused by Ca deficiency. Spray application of Ca alleviates the occurrence of cork spot disorder in pear (Faust and Shear, 1969; Richardson and Lombard, 1979; Raese and Drake, 1993; Raese and Drake, 1995; Raese and Drake, 2006). In addition, certain mineral nutrients affect Ca effectiveness in fruit (Lee et al., 2007); for example, potassium (K) and magnesium (Mg) can antagonize the absorption or functioning of Ca on the cell membrane or compete for the binding sites of Ca on the membrane (Schönherr and Bukovac, 1973; Yermiyahu et al., 1994). A high content of nitrogen (N) in the fruit aggravates the Ca deficiency symptoms in apple (Shear, 1971; Bangerth, 1974). Higher contents of N and Mg, and lower contents of K and phosphorus (P), are reported in cork-spotted pear fruit. Therefore, not only the Ca content but also the balance between Ca and other mineral nutrients should be considered as possible causal factors for cork spot disorder. Notably, alternative conclusions have been advanced in certain studies of the relationship between Ca nutrition and cork spot disorder. For example, no significant difference in Ca content has been observed between cork-spotted fruit and non-affected fruit, and higher concentrations of stored Ca and free Ca^2+^ have been detected in some cork-spotted fruit; in addition, the ratios of K/Ca, Mg/Ca, and (K+Mg)/Ca contents in cork-spotted fruit are significantly lower than those in non-affected fruit (Mason and Welsh, 1970; Duan et al., 2019). Therefore, the Ca concentration in the fruit is not a reliable indicator for monitoring the incidence of cork spot disorder in pear.

**Figure 4:**
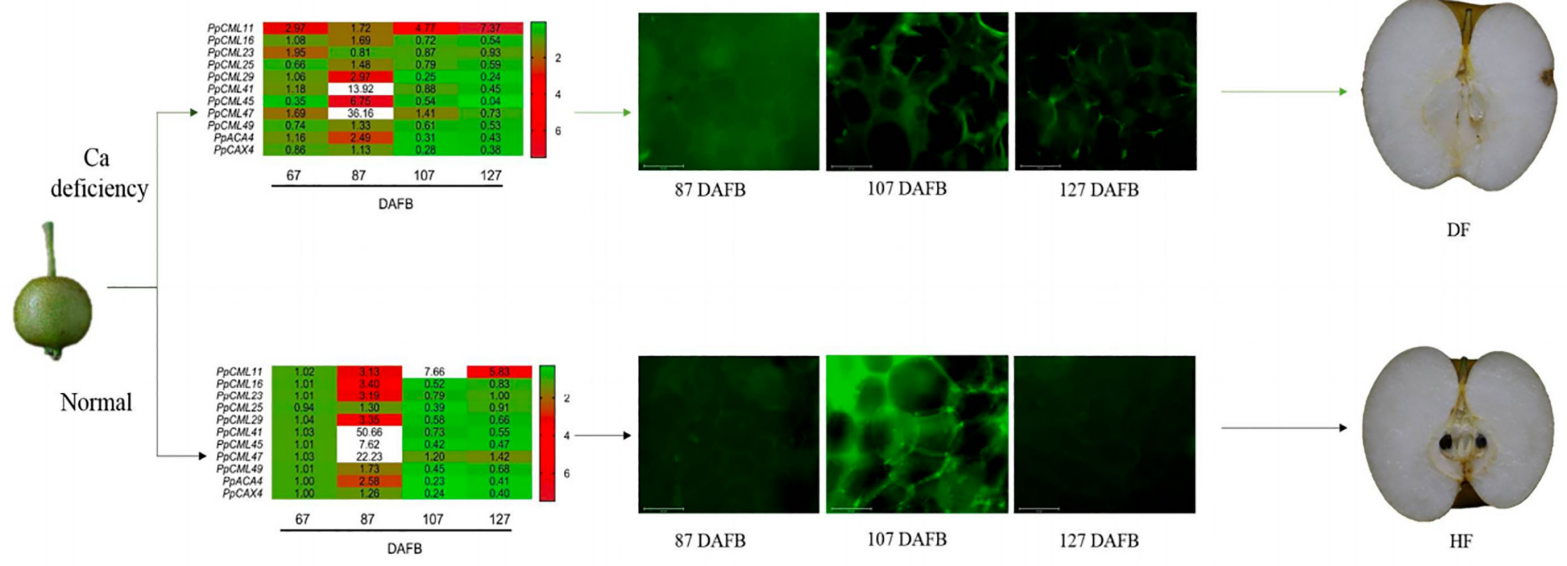
A hypothetical effect of the gene expression and Ca^2+^ distribution on the incidence of cork spot disorder in ‘Akizuki’ (Cui et al., 2021b). Nitrendipine treatment caused the Ca deficiency in the flesh at an early stage of the fruit development (67 DAFB). Expression levels of Ca^2+^ signal transduction-related genes were then altered, which further promoted the intracellular imbalance of Ca^2+^ distribution and promoted the cork spot disorder development. Green fluorescence indicated the distribution of Ca^2+^. DF means cork spot disordered fruit; HF means healthy fruit.

The relationship between Ca deficiency and incidence of cork spot disorder requires further exploration. Observations on the intracellular distribution of Ca in cork-spotted fruit have shown that Ca^2+^ is concentrated within the cell wall and is less abundant in the cytoplasm. The effect of the uneven intracellular distribution of Ca^2+^ on the occurrence of cork spot disorder needs further study as shown in **Figure 4**. Both long-distance and transmembrane transport affect the Ca^2+^ distribution in the fruit, and browning and necrosis of the vascular tissues are observed in cork-spotted pear fruit (Cui et al., 2021a). Therefore, dysfunction of the vascular bundles in the fruit may be responsible for the uneven distribution of Ca^2+^, suggesting that damage to the vascular tissues may precede the initiation of cork spot disorder (Cui et al., 2021a). Gene expression analysis suggests that Ca^2+^ transport-related genes also play important roles in the initiation of cork spot disorder (Cui et al., 2021a). Nitrendipine (a Ca-uptake inhibitor) treatment reduces the Ca content during early fruit development, changes the expression levels of Ca^2+^ transport-related genes, affects the intracellular distribution of Ca^2+^ in the fruit, and ultimately promotes the incidence of cork spot disorder (Cui et al., 2021b). In addition, during the growing season, Ca^2+^ can be transferred from the fruit to the leaves and shoots, which may lead to an imbalanced distribution of Ca^2+^ in the fruit and increase the incidence of bitter pit in apple (Vang-Petersen, 1980). Thus, preventive measures should be taken at an early stage of fruit development, especially at the critical stage for initiation of cork spot disorder.

#### 3.4.2 Boron

Boron (B) affects the occurrence of bitter pit in apple (Jemrić et al., 2016), but no consistent conclusion has been drawn on the relationship between B content and incidence of cork spot disorder in pear fruit. The B content is higher in cork-spotted fruit than in non-affected fruit of ‘Akizuki’ (Matsuda and Yamanouchi, 2018). Quantification of B contents in the soil, leaves, and fruit has shown that, under B deficiency in the soil, no similar trend is observed in the leaves. In addition, no significant correlation between the B content in the fruit and incidence of cork spot disorder was observed. Thus, the effect of B application to the soil on cork spot disorder is inconclusive (Uemura et al., 2009). Boron is less essential for fruit growth and development, and the pivotal time to supply is the bloom phase. In China, application of B to the soil and as a foliar spray is relatively common in some pear orchards to prevent cork spot disorder. However, further investigation of the effectiveness of B in preventing cork spot disorder is needed.

### 3.5 Plant growth regulators

Gibberellins are widely applied in Asian pear production to promote fruit growth by smearing on the fruit peduncle. Given the lower number and larger size of cells in the flesh of GA-treated fruit (Hayama et al., 2017; Mitani et al., 2017; Lv, 2021), the fruit enlarges more rapidly in response to GA treatment than in non-treated fruit during the fruit enlargement phase, which results in deficiencies in the nutrient status of the fruit. Therefore, treatment of Asian pear with GA significantly increases the fruit size, decreases the fruit sugar content, increases the fruit pH, and markedly increases the incidence of cork spot disorder (Hayama et al., 2017). The incidence of cork spot disorder is strongly associated with GA-treated fruit, especially with large fruit. Therefore, GA should be used appropriately and carefully to control fruit size, so as to achieve a balance between the increase in fruit yield and promote the occurrence of cork spot disorder. Application of *N*-(2-chloro-4-pyridinyl)-*N*-phenylurea to pear fruit increases the fruit cell number but decreases the fruit cell size and incidence of cork spot disorder (Tamura, 2017). Spray application of ethephon at 100 DAFB promotes early ripening and reduces the occurrence of cork spot disorder in ‘Akizuki’ fruit, whereas ethephon treatment reduces the incidence of cork spot disorder but causes severe watercore disorder in ‘Oushuu’ fruit (Mitani et al., 2017; Hiramoto and Kitamura, 2020).

### 3.6 Environmental factors

Pear tree growth is influenced by climatic factors, such as light, temperature, and relative humidity, as well as severe weather events, such as low temperature, frost, and hail. Pear cork spot disorder is a fruit physiological disorder associated with nutritional stresses. The study of this disorder cannot be separated from environmental factors, which play important roles in the overall nutrient balance of the tree. Our field investigations have shown that the incidence of cork spot disorder in pear fruit may be as low as 5% or as high as 70% in different years in the same orchard under the same management practices. This finding demonstrates that environmental factors critically influence the incidence of cork spot disorder in pear.

#### 3.6.1 Light

Light directly affects leaf transpiration and photosynthesis, and plays an important role in the nutritional status and metabolism of pear trees. Leaf transpiration is enhanced under high-intensity light, promoting the transport of water and nutrients from the roots to the leaves and fruit. However, strong light can also cause stomatal closure and leaf withering, and the absorption of certain nutrients may be depressed, leading to the occurrence of physiological disorders in the fruit. The incidence of cork spot disorder differs between bagged and non-bagged fruit, and tree shading increases the incidence of cork spot disorder (Matsumoto et al., 2018). In addition, Ca^2+^ transport to the fruit predominantly occurs to young fruit. Fruit Ca^2+^ import is strongly associated with the light environment and increases under strong light and decreases under weak light (Liu et al., 2005). These phenomena indicate that there is a close relationship between light and Ca^2+^ metabolism in the fruit, but the mechanism requires further study.

#### 3.6.2 Temperature

Temperature is an important factor that influences the incidence of cork spot disorder in pear. High temperature in summer promotes competition for water between the leaves and the fruit, which intensifies water stress on the fruit and increases the incidence of cork spot disorder (Tamura, 2017). Based on investigation over several years, a high temperature in July and August is critical for the development of cork spot disorder in ‘Akizuki’ fruit and significantly increases the incidence of cork spot disorder (Wang, 2021). In addition, dry weather in July reduces the Ca content in leaves and Ca absorption from dry soil is inhibited (Shimada et al., 2018). Therefore, measures should be implemented to alleviate high-temperature stress on trees in an orchard in summer, which may affect nutrient metabolism in the fruit and promote the occurrence of cork spot disorder.

#### 3.6.3 Soil pH

The physicochemical properties of the soil affect nutrient absorption by the tree, and the cork spot disorder in pear can be alleviated by increasing the soil organic matter content (Wang, 2021). However, under the same management practices, the incidence of cork spot disorder is significantly higher in trees growing in alkaline soil with a high pH than in trees growing in acidic soil with a low pH (Wang, 2021). For example, in Hebei, Anhui, and Shandong provinces and other locations in China, the incidence of cork spot disorder in an orchard with alkaline soil is significantly higher than in an orchard with non-alkaline soil. Under an alkaline soil pH, the absorption of some nutrients may be limited, leading to an imbalance in nutrient supply and promotion of cork spot disorder. However, the specific nutrients that are affected in alkaline soil remain unclear and require investigation.

### 3.7 Cultivation management

#### 3.7.1 Tree growth control

Tree vigor has an important impact on fruit production. Based on a survey of Japanese pear orchards, trees that show vigorous growth and produce a higher number of water sprouts have a higher incidence of cork spot disorder (Nakamura, 2011; Tamura, 2017). The incidence of cork spot disorder varies among scaffold branches on the same tree of the Chinese white pear ‘Chili’; cork spot disorder is more frequently observed on a strong branch, whereas a lower incidence is observed on a weak or moderate branch. The incidence of cork spot disorder decreases with increase in stability of branch growth. In addition, young trees show a higher incidence of cork spot disorder than older trees. Interestingly, trees that show normal cessation of growth of the new shoots in summer exhibit a lower incidence of cork spot disorder than that of trees without normal growth cessation (Wang, 2021). These results indicate that maintenance of stable growth and control of the growth rhythm of the tree, and coordination of the balance between vegetative growth and reproductive growth, are beneficial for the stable supply of nutrients to the fruit and can reduce the incidence of cork spot disorder.

#### 3.7.2 Floral bud selection

The floral bud types in pear mainly comprise axillary, spur, and clustered spur buds. The fruit that develop from the different types of floral bud show differences in fruit nutrient supply, which may affect the incidence of cork spot disorder. In pear, the fruit that develop from different types of floral bud differ in the incidence of cork spot disorder (Xu, 2017). Pear flowers are borne in umbels, generally of six to eight flowers, of which the lateral flowers open first. The growth rate of fruit developing in different positions of the inflorescence varies. The fruit developing from lateral flowers and central flowers differ significantly in size, shape, and timing of maturity (Xu, 2017). Large fruit show a higher incidence of cork spot disorder; therefore, removal of the fruit that develop from lateral flowers, which generally give rise to large fruit, could be imposed to reduce the incidence of cork spot disorder. In addition, early fruit thinning is beneficial to preventing the occurrence of cork spot disorder (Hiramoto and Kitamura, 2020). Early fruit thinning leads to an adequate nutrient supply to the remaining immature fruit, which increases the number of fruit cells, avoids an excessive cell size at advanced stages of fruit development, and decreases the incidence of cork spot disorder.

#### 3.7.3 Fertilizer and water management

Excessive application of N in a pear orchard promotes cork spot disorder, but N deficiency is unfavorable for fruit growth (Shimada et al., 2013). Therefore, suitable application of N, based on the tree N demand in the growing season, is crucial. At different time points in the growing season, Ca supplementation in the soil and as a foliar spray may reduce the occurrence of cork spot disorder. During fruit development in ‘Akizuki’, the contents of N, Ca, Mg, and zinc show a decreasing trend whereas the contents of P and K initially decrease and thereafter increase (Jiao, 2019). The ratios of K/Ca, Mg/Ca, and (K+Mg)/Ca increase sharply in July. The contents of soluble sugar, reducing sugar, and ascorbic acid increase gradually, whereas the contents of starch, cellulose, and protopectin, and the activities of cellulase and polygalacturonase initially increase and thereafter decrease in July (Jiao, 2019). These findings indicate that the fruit rapidly enlarges at approximately 70 DAFB, coinciding with substantial changes in the nutrient content of the fruit, and thus it is a sensitive period for the incidence of cork spot disorder. Therefore, a stable water and nutrient supply during July is important to prevent the development of cork spot disorder.

#### 3.7.4 Harvest time

The timing of the onset of cork spot disorder varies among pear cultivars. In most cultivars, cork spot disorder is initiated during the fruit enlargement phase, and the cork spot symptoms gradually increase in severity with the progression of fruit development until harvest. Therefore, early harvesting can mitigate the severity of cork spot disorder symptoms and mitigate the reduction in commodity value of the fruit (Hayama et al., 2017). In addition, during postharvest storage, cork spot disorder symptoms remain unchanged, especially under storage at low temperature, and the respiratory metabolism rate of the fruit is low and does not aggravate the symptoms (Wang, 2021). These findings indicate that cork spot disorder is initiated during fruit development and is closely associated with orchard management and environmental factors, and thus differs from other physiological disorders arising during postharvest storage.

### 3.8 Gene expression alteration

Although the first report of pear cork spot disorder was early enough (McAlpine, 1921), most of the studies have been focusing on the histological and biochemical analyses of the cork spotted fruits since then. In recent years, we investigated the effect of gene expression alteration on the incidence of cork spot disorder (Cui et al., 2021a; Cui et al., 2021b). The results showed that gene expression alteration happened as early as 67 DAFB, prior to the initiation of cork spot disorder. Most of the genes with altered expression belonged to Ca^2+^-transported and Ca^2+^ signal transduction-related families (e.g., *CNGCs*, *CAXs*, *ACAs*, and *CMLs*). This indicated that Ca^2+^ metabolism affected the initiation and development of cork spot disorder through altering the Ca^2+^ transport, Ca^2+^ signal transduction, and Ca^2+^ distribution in the fruit. More details of the molecular mechanism of the occurrence of cork spot disorder need further digging.

## 4 Preventive measures for cork spot disorder

### 4.1 Maintenance of stable tree growth

Based on investigations in the field, the fruit born on strong branches show a higher incidence of cork spot disorder than fruit born on moderate branches, and young trees show a higher incidence of cork-spotted fruit than older trees (Jiao, 2019). These findings indicate that incidence of cork spot disorder is closely associated with the stability of tree growth and that an older tree and moderate branches provide stable water and nutrient supply to the fruit, thereby reducing the occurrence of cork spot disorder. Therefore, it is crucial to maintain the stability of tree growth by restricting excessive growth of young trees, controlling the growth of vigorous trees, and counterbalancing shoot growth and fruiting by summer pruning, which are effective measures to reduce the incidence of cork spot disorder (Cui et al., 2020).

### 4.2 Maintenance of stable fertilizer and water supply

It is important of avoid irregular fertilizer and water supply, which cause unstable developmental rhythms of the fruit, especially during the periods of rapid growth of the new shoots and fruit enlargement (Xu, 2017). Therefore, maintaining stable fertilizer and water supply according to the demands of the tree and coordinating the competition for water and nutrients between the shoots and the fruit are essential to controlling the occurrence of cork spot disorder.

### 4.3 Improvement of soil properties

The orchard soil physicochemical properties can be improved by application of an organic fertilizer, which enhances the soil retention capacity for water and mineral elements (Xu, 2017). In addition, an organic fertilizer is important to prevent the soil from becoming excessively dry in summer and to maintain the soil pH at 5–7, which is beneficial to preventing the occurrence of cork spot disorder (Xu, 2017).

### 4.4 Prevention of high-temperature stress in summer

Cork spot disorder of pear is strongly affected by environmental factors, especially high temperature in summer, which alters the growth rate of tree branches and roots and changes the capacity of the tree for nutrient supply to the fruit (Wang, 2021). In addition, the adsorption of certain nutrients is restricted under high-temperature stress in summer. Installation of a hanging sprinkler system in a pear orchard would regulate the temperature and relative humidity in summer, which is beneficial to maintaining stable tree growth and nutrient supply, and reduce the incidence of cork spot disorder (Wang, 2021).

## 5 Future research

**Figure 5:**
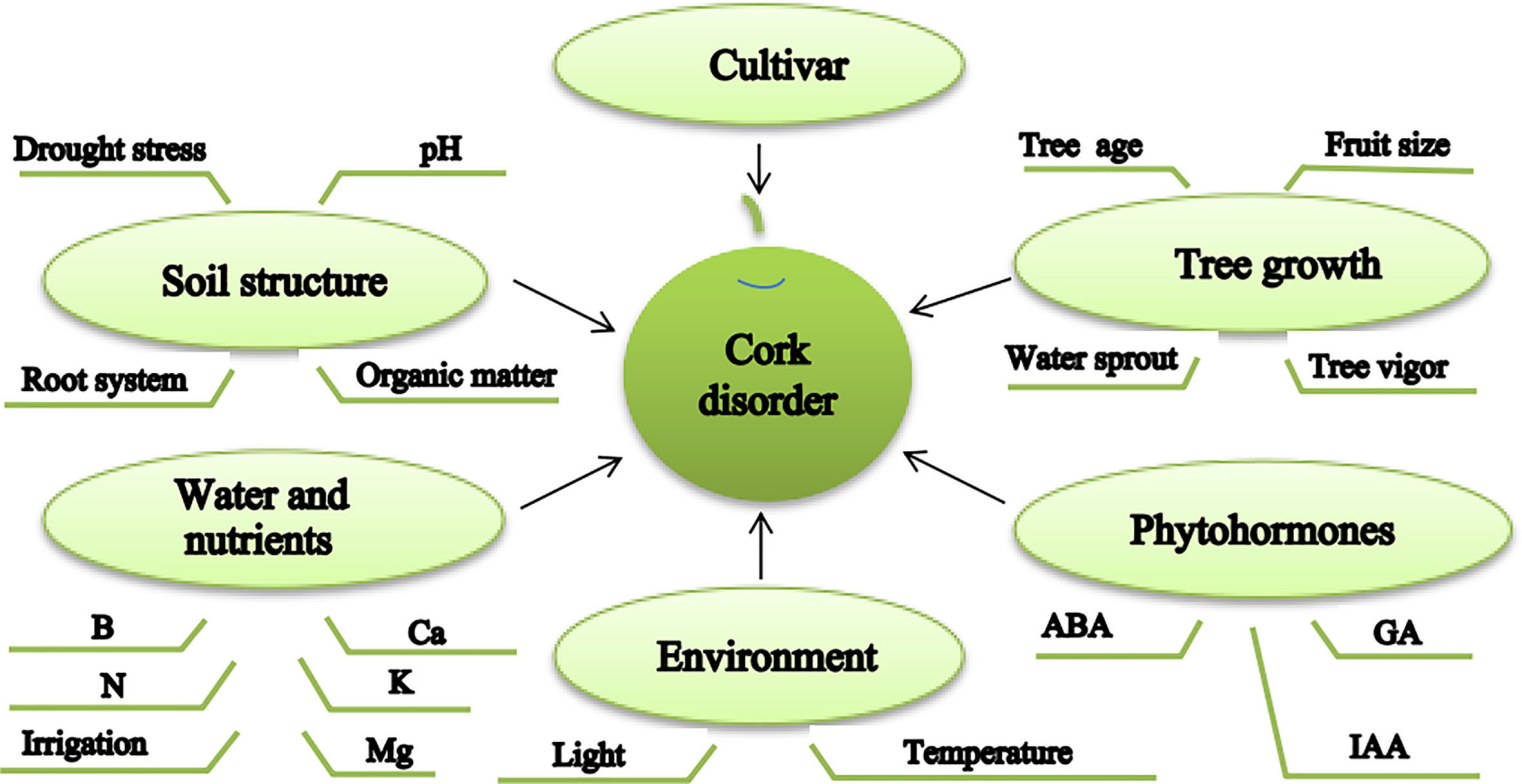
Schematic diagram of the causal factors of cork spot disorder in fruit of Asian pear.

Although some previous research has been conducted, considerable information regarding the mechanism of cork spot disorder remains to be elucidated. Effective measures to prevent cork spot disorder in pear production require improvement. It is evident that cultivar specificity, cultivation practices, environmental factors, and soil properties all affect the incidence of cork spot disorder (**Figure 5**). In future research, the mechanism of cork spot disorder should be explored at the molecular level using multiomics tools, which is crucial for the breeding of new cultivars with improved tolerance of factors responsible for cork spot disorder.

## Author contributions

XZ provided the idea and prepared the manuscript; ZC revised the manuscript. All authors contributed to the article and approved the submitted version.

## References

[B1] Bangerth F. (1974). Antagonism between calcium and other elements in the apple fruit. Acta Hortic. 45, 49–52.

[B2] Cui Z. H. Jiao Q. Y. Wang R. Ma C. H. (2020). Investigation and analysis of relationship between mineral elements alteration and cork spot physiological disorder of Chinese pear ‘Chili’ (*Pyrus bretschneideri* rehd.). Sci. Hortic. 260, 108883. doi: 10.1016/j.scienta.2019.108883

[B3] Cui Z. H. Wang N. N. Duan Y. X. Xu X. R. Wang R. Zhang S. L.. (2021a). High-resolution microstructure analysis of cork spot disordered pear “Akizuki” (*Pyrus pyrifolia* nakai) using X-ray CT. Front. Plant Sci. 12, 715124. doi: 10.3389/fpls.2021.715124 34484278 PMC8415714

[B4] Cui Z. H. Wang N. N. Li D. L. Wang R. Ma C. H. (2021b). Nitrendipine-treatment increases cork spot disorder incidence in pear ‘Akituki’ (Pyrus pyrifolia nakai.) by altering calcium distribution inside the fruit. Plants 10, 994. doi: 10.3390/plants10050994 34067594 PMC8155913

[B5] Dong Y. Guan J. F. Ma S. J. Liu L. L. Feng Y. X. Cheng Y. D. (2015). Calcium content and its correlated distribution with skin browning spot in bagged *Huangguan* pear. Protoplasma 252, 165–171. doi: 10.1007/s00709-014-0665-5 24965371

[B6] Dong X. C. Wang H. W. Wei S. W. Ran K. Dong R. Wang S. M. (2018). Causes of flesh browning by suberification in pear fruit and its control in midwest Shandong province. J. Fruit Sci. 35 (Suppl.), 139–142.

[B7] Duan Y. X. Xu Y. Wang R. Ma C. H. (2019). Investigation and prevention of cork spot disorder in ‘Akizuki’ pear (*Pyrus pyrifolia* nakai). HortScience 54, 480–486. doi: 10.21273/HORTSCI13775-18

[B8] Duan Y. X. Xu X. R. Wang R. Ma C. H. (2020). X-Ray μCT analysis of cork spot disorder in Chinese pear ‘Chili’ (*Pyrus bretschneideri*). Postharvest Biol. Technol. 170, 111321. doi: 10.1016/j.postharvbio.2020.111321

[B9] Faust M. Shear C. B. (1969). Biochemical changes during the development of cork spot of apples. Qual. Plant Mater. Veg. 19, 255–265. doi: 10.1007/BF01101157

[B10] Hayama H. Mitani N. Yamane T. Inoue H. Kusaba S. (2017). Characteristics of cork spot like disorder in Japanese pear ‘Akizuki’ and ‘Oushuu’. Hortic. Res. (Japan) 16, 79–87. doi: 10.2503/hrj.16.79

[B11] Hiramoto M. Kitamura M. (2020). Early fruit thinning and treatment with ethephon shows synergy effects to alleviate cork-spot-like disorder in Japanese pear ‘Akizuki’. Res. Bull. Kumamoto Prefect. Agric. Res. Cent. 27.

[B12] Jemrić T. Fruk I. Fruk M. Radman S. Sinkovič L. Fruk G. (2016). Bitter pit in apples: pre- and postharvest factors: a review. Span. J. Agric. Res. 14, e08R01. doi: 10.5424/sjar/2016144-8491

[B13] Jiao Q. Y. (2019). Physiological and molecular biological analysis of cork spot like disorder in laiyang ‘Chili’ (Pyrus bretschneideri rehd) (Qingdao: MSc Dissertation, Qingdao Agricultural University).

[B14] Kajiura I. Yamaki S. Omura M. Shimura I. (1976). Watercore in Japanese pear (*Pyrus serotina* rehder var. ‘Culta’ rehder). i. description of the disorder and its relation to fruit maturity. Sci. Hortic. 4, 261–270. doi: 10.1016/0304-4238(76)90049-2

[B15] Lee S. H. Choi J. H. Kim W. S. Park Y. S. Gemma H. (2007). Effects of calcium chloride spray on peroxidase activity and stone cell development in pear fruit (*Pyrus pyrifolia* ‘Niitaka’). J. Jpn. Soc Hortic. Sci. 76, 191–196. doi: 10.2503/jjshs.76.191

[B16] Liu X. Y. Shi H. W. Li L. (2005). Influence of light intensity on the change of Ca^2+^ and Zn^2+^ in the growth of dangshansu pear. J. Anqing Teachers Coll. (Nat. Sci.) 11 (4), 50–51.

[B17] Lv Y. L. (2021). Analysis of pathogenic factors and prevention of cork spot disorder of ‘Akizuki’ pear. Hebei For. 33.

[B18] Ma C. H. Li D. L. Wang R. (2014). Microscopic observation of the ‘hard-end disorder’ on the pulp tissue of ‘Hwangkumbae’. North. Hortic. 13, 109–113.

[B19] Mason J. L. Welsh M. F. (1970). Cork spot (pit) of ‘Anjou’ pear related to calcium concentration in fruit. HortScience 5, 447. doi: 10.21273/HORTSCI.5.5.447

[B20] Matsuda K. Yamanouchi D. (2018). Cultivation environment involved in the occurrence of flesh browning disorder of Japanese pear ‘Akizuki’. Hortic. Res. (Japan) 2, 165.

[B21] Matsumoto K. Kobayashi T. Kougo T. Fujita T. Sato S. Moriguchi T. (2018). Prevention of new cork spot-like physiological disorder in ‘Kurenainoyume’ apples by pre-harvest fruit bagging. Hortic. J. 87, 174–183. doi: 10.2503/hortj.OKD-117

[B22] McAlpine D. (1921). Bitter pit on apple and pears. the latest results in preservation measures. Phytopathology 11, 366–370.

[B23] Miqueloto A. Talamini do Amarante C. V. Steffens A. D. Santos A. Mitcham E. (2014). Relationship between xylem functionality, calcium content and the incidence of bitter pit in apple fruit. Sci. Hortic. 165, 319–323. doi: 10.1016/j.scienta.2013.11.029

[B24] Mitani N. Hayama H. Yamane T. Kusaba S. (2017). Effects of flower position, flowering time, and ethephon treatment, which affect the fruit maturation time, on the cork spot-like disorder of Japanese pear ‘Akizuki’ and ‘Oushuu’. Hortic. Res. (Japan) 16, 471–477. doi: 10.2503/hrj.16.471

[B25] Nakamura Y. (2011). Flesh disorder investigation report in Japanese pear fruit ‘Akizuki’ and ‘Oushuu’. Bull. Natl. Inst. Fruit Tree Sci. 12, 33–63.

[B26] Raese J. T. (1988). Calcium sprays and fertilizers found effective against ‘d’Anjou’ pears disorders. Good Fruit Grower 39, 35–39.

[B27] Raese J. T. Drake S. R. (1993). Effects of preharvest calcium sprays on apple and pear quality. J. Plant Nutr. 16, 1807–1819. doi: 10.1080/01904169309364651

[B28] Raese J. T. Drake S. R. (1995). Calcium sprays and timing affect fruit calcium concentrations, yield, fruit weight, and cork spot of ‘d’Anjou’ pears. HortScience 30, 1037–1039. doi: 10.21273/HORTSCI.30.5.1037

[B29] Raese J. T. Drake S. R. (2006). Calcium foliar sprays for control of alfalfa greening, cork spot, and hard end in ‘Anjou’ pears. J. Plant Nutr. 29, 543–552. doi: 10.1080/01904160500526683

[B30] Richardson D. G. Lombard P. B. (1979). Cork spot of anjou pear: control by calcium sprays. Commun. Soil Sci. Plant Anal. 10, 383–389. doi: 10.1080/00103627909366902

[B31] Schönherr J. Bukovac M. J. (1973). Ion exchange properties of isolated tomato fruit cuticular membrane: exchange capacity, nature of fixed charges and cation selectivity. Planta 109, 73–93. doi: 10.1007/BF00385454 24473974

[B32] Shear C. B. (1971). Symptoms of calcium deficiency on leaves and fruits of ‘York imperial’ apple. J. Am. Soc Hortic. Sci. 96, 415–417. doi: 10.21273/JASHS.96.4.415

[B33] Shimada T. Katano T. Ohba E. Inoue H. Hayama Y. Yamane T. (2013). Effects of fertilizer application on pulp damage in Japanese pear ‘Akizuki’. Hortic. Res. (Japan). 12 (47).

[B34] Shimada A. Tominaga S. Yamamoto M. (2018). Effects of water management on vine growth and fruit quality in passion fruit. Hortic. Res. (Japan). 17, 1–10. doi: 10.2503/hrj.17.1

[B35] Tamura F. (2017). Occurrence of physiological disorders in Japanese pear fruit and advances in research on these disorders. Hortic. Res. (Japan). 16, 373–381. doi: 10.2503/hrj.16.373

[B36] Tamura F. Chun J. P. Tanabe K. Morimoto M. Itai A. (2003). Effect of summer-pruning and gibberellin on the watercore development in Japanese pear ‘Akibae’ fruit. J. Jpn. Soc Hortic. Sci. 72, 372–377. doi: 10.2503/jjshs.72.372

[B37] Tanabe K. Hayashi S. Murayama N. (1982). Relationships between degrees of occurrence of “Yuzuhada” disorder and contents of inorganic elements of fruit pulp in Japanese pear cultivars. J. Jpn. Soc Hortic. Sci. 50, 432–435. doi: 10.2503/jjshs.50.432

[B38] Uemura H. Kaku R. Okada S. Nakamitsu K. Iwatani A. Sakaki H. (2009). Actual occurrence of flesh disorders of Japanese pear ‘Akizuki’ in kumamoto prefecture. Hortic. Res. (Japan). 1, 50.

[B39] Vang-Petersen O. (1980). Calcium nutrition of apple trees: a review. Sci. Hortic. 12, 1–9. doi: 10.1016/0304-4238(80)90032-1

[B40] Wang N. N. (2021). Impact of calcium metabolism on the development of cork spot physiological disorder of pear ‘Akizuki’ (Pyrus pyrifolia nakai) (Qingdao: MSc Dissertation, Qingdao Agricultural University).

[B41] Wang L. Jia J. A. Zhu X. W. Ding G. Z. Pei Z. L. (1990). Regulation and prevention of browning of fruit pulp of ‘Zaosu’ pear. China Fruits. 1, 35–36.

[B42] Xu Y. (2017). The effect of fruit development and nutrient metabolism on cork spot disorder in the ‘Akizuki’ pear (Pyrus pyrifolia nakai) (Qingdao: MSc Dissertation, Qingdao Agricultural University).

[B43] Yamamoto T. Watanabe S. (1982). Initial time of development of hard end disorder in ‘Bartlett’ pear. J. Jpn. Soc Hortic. Sci. 51, 142–151. doi: 10.2503/jjshs.51.142

[B44] Yermiyahu U. Nir S. Ben-Hayyim G. Kafkafi U. (1994). Quantitative competition of calcium with sodium or magnesium for sorption sites on plasma membrane vesicles of melon (*Cucumis melo* l.) root cells. J. Membr. Biol. 138, 55–63. doi: 10.1007/BF00211069 8189432

